# Human Dental Pulp Stem Cells Exhibit Osteogenic Differentiation Potential

**DOI:** 10.1515/biol-2020-0023

**Published:** 2020-05-06

**Authors:** Sadia Awais, Samira Shabbir Balouch, Nabeela Riaz, Mahmood S Choudhery

**Affiliations:** 1 Tissue Engineering and Regenerative Medicine Laboratory, Department of Biomedical Sciences, King Edward Medical University, Lahore, Pakistan; 2 Department of Oral and Maxillofacial Surgery, King Edward medical University, Lahore, Pakistan

**Keywords:** human dental pulp stem cells, impacted tooth, third molars, dental pulp, osteoblast

## Abstract

Bone regeneration after trauma, pathologic and surgical procedures is considered a major medical challenge. Due to limitations in using conventional approaches, cell based regenerative strategies may provide an alternative option to address such issues. In the current study, we sought to determine the osteogenic potential of dental pulp stem cells (DPSCs) isolated from impacted 3^rd^ molars. DPSCs were isolated from human dental pulp tissue (n=6) using explant culture. Growth characteristics of DPSCs were determined using plating efficiency, and the number and time of population doublings. After characterization, DPSCs were induced to differentiate into osteoblasts and were assessed using polymerase chain reactions (PCR) and histological analysis. Results indicated that DPSCs can be isolated from impacted human third molars, and that DPSCs exhibited typical fibroblastic morphology and excellent proliferative potential. In addition, morphological changes, histological analysis and expression of lineage specific genes confirmed osteogenic differentiation of DPSCs. In conclusion, DPSCs isolated from impacted 3rd molars have high proliferative potential and ability to differentiate into osteoblasts.

## 1 Introduction

The refinement of bone imperfection and defects due to trauma, pathological conditions and surgical procedures is a significant challenge [1]. Currently, allografts, autologous bone grafts or alloplastic materials are used to overcome the above-mentioned defects. However, certain limitations such as inadequate quantity of grafting bones, deformity, donor site morbidity, poor biocompatibility, immunogenicity and compromised vascularity restrict their use for optimum clinical conditions [1, 2]. Cell-based tissue engineering strategies can offer unique therapeutic alternative approaches to address these issues. Isolation of stem cells (SCs) from human adult and deciduous teeth has been reported in the last decade [3]. Adult stem cell sources, such as dental pulp (a soft living tissue within teeth) is a source of MSC (mesenchymal stem cells) like cells and may be used for the repair of large bone defects in dentoalveolar and craniofacial regions [4, 5, 6, 7, 8]. It has been observed that mesenchymal stem cells (MSCs) can differentiate into bone-like cells when the appropriate environment is provided. As dental pulp stem cells (DPSCs) exhibit MSC like characteristics, we aimed to isolate human dental pulp stem cells (hDPSCs) from the impacted 3^rd^ molar and induce them to differentiate into osteoblasts.

The use of DPSCs can be very cost-effective, less invasive, convenient, and safer with fewer complications and no ethical issues as compared with other MSCs sources such as bone marrow, peripheral blood, and umbilical cord blood etc. [9]. These cells can easily be cultivated and expanded for autologous, as well as allogenic medical use. Further, these cells can be cryopreserved, are immunoprivileged and also when grafted into allogenic tissues exhibit anti-inflammatory abilities. Highly proficient interaction with biomaterials makes it a favorable choice for bone engineering [10].

In oral, and maxillofacial surgery, the restoration of function, such as facial expression, occlusion and mastication is exquisitely complex and the unconventional possibilities of enhancing bone regeneration should be considered [11, 12, 13]. Since MSCs can be induced to differentiate into osteoblasts, the current study aims to evaluate the osteogenic potential of DPSCs which exhibit MSC-like characteristics and is a new unconventional source of stem cells. In addition, cryopreservation of DPSCs will allow use of these autologous cells at the time of care when needed [14, 15].

The present study is intended to assess the osteogenic potential of MSCs harvested from dental pulp of extracted impacted 3^rd^ molar. DPSCs were isolated from the pulp using an explant culture method. Cells were characterized, and their proliferation was determined using parameters such as plating efficiency, population doubling time and population doublings. DPSCs were then induced to differentiate into osteoblasts and their differentiation was assessed using various parameters. Results indicated that dental pulp of 3^rd^ molars can be used for isolation of stem cells that are highly proliferative and demonstrate osteogenic potential. This study will provide opportunity for the use of extracted teeth as an autologous cell source for patient care to restore bone defects.

## 2 Material and methods

This *in vitro* experimental study was conducted in the Tissue Engineering and Regenerative Medicine Laboratory, Department of Biomedical Sciences at King Edward Medical University/Mayo Hospital Lahore. Six samples of human healthy third molars were removed for orthodontic or prophylactic measures in the Oral and Maxillofacial Surgery Department of Mayo Hospital Lahore. Wisdom teeth with deep caries, periapical and periodontal disease, sclerosed pulp chamber or donors positive for HCV, HBV and HIV were excluded from study.

**Informed consent**: Informed consent has been obtained from all individuals included in this study.

**Ethical approval**: The research related to human use has been complied with all the relevant national regulations, institutional policies and in accordance the tenets of the Helsinki Declaration, and has been approved by the Institutional Review Board (IRB) and AS&RB (Advance Studies and Research Board) at King Edward Medical University, Lahore. The ethical approval letter is #188/ RC/ KEMU.

### 2.1 Isolation and Culturing of DPSCs

Before extraction of healthy impacted 3rd molars, each subject was evaluated for systemic and oral infections. Immediately after extractions, the tooth was placed in phosphate buffer saline (PBS) supplemented with 1% penicillin (100U/ml) and streptomycin (100ug/ml). Before the extraction of pulp, molars were washed with ethanol (70%). An incision was made at the enamel and cementum junction using a cylindrical turbine bur. Pulp from tooth was removed with sterilized forceps or barbed brochae [6]. Pulp tissue was then washed with PBS and minced into 1–2 mm fragments. Tissue fragments were cultured in α-MEM (Minimum Essential Medium-Alpha, Caisson MEL07-500ML) supplemented with 1% non-essential amino acids (Caisson cat# 02161008), 1% penicillin/streptomycin solution (Capricorn PS-B, cat# CP13-1019), 1% L-glutamine (Hyclone cat# SH30034.01), 1% sodium pyruvate (Hyclone cat# SH30239.01) and 10% fetal bovine serum (FBS, Biochorm cat# 0878C). Six-well plates containing tissue pieces were incubated at 37°C, and 5% CO_2_ in humid environment. After 24 hours, the medium was replaced and cell cultures were observed daily. When a sufficient number of colonies was observed, tissue pieces were removed and fresh medium was added. When cultures reached 80–90% confluence, they were dissociated using trypsin-EDTA, counted and plated for subsequent experiments. DPSCs, like MSCs were defined by their fibroblastic morphology and plastic adherent properties [16].

### 2.2 Growth Characteristics of Dental Pulp Stem Cells

Growth characteristics of DPSCs were determined by using parameters such as plating efficiency (PE), number of population doublings (PD) and population doubling time (PDT).

### 2.3 Plating Efficiency (PE)

To determine plating efficiency, DPSCs at passage 1 were plated in low numbers as described by Choudhery et al [15]. Briefly, passage 1 DPSCs were trypsinized and counted using a hemocytometer. DPSCs were plated at a concentration of 40 cells per cm^2^ (1000 cells per 25 cm^2^ culture flasks). Complete culture medium was added, and cultures were incubated at 370C at 5% CO_2_ under humid conditions. After two weeks, absolute methanol was used to fix resultant colonies. Colonies of cells were then stained with 0.1% crystal violet dye, and counted. The following formula was used for measuring the plating efficiency [15]:

Plating Efficiency (P.E.) = (No. of colonies counted/ No. of cells initially plated) × 100

### 2.4 Population Doubling and Doubling Time

The number of population doublings was determined by passaging the cells serially at a 1:10 dilution. Initial and final cell numbers were determined at each passage for up to ten passages. Population doublings (PD) and population doubling time (PDT) was calculated using the following formulae [14, 15]:

$$\begin{array}{rl} & \text{cPDs}\;\;=\;\text{Log}\;\text{N/}\;\;\text{No}\times \log\;\;2\\ & \;\;\;\;\;\;\;\;\;\text{DT}\;\text{=}\;\text{CT/}\;\text{cPDs} \end{array}$$

Where, “cPDs” represents cumulative population doublings, and “No” represents cell number plated, “N” represents cell number harvested, “DT” is doubling time and “CT” is total time in culture.

### 2.5 Osteogenic differentiation of DPSCs

For *in vitro* osteogenic differentiation, passage two DPSCs at a concentration of 5×10^4^ cells were plated in 6-well tissue culture plates with complete expansion culture media. When DPSCs cultures became 80%-90% confluent, the primary culture medium was replaced by osteogenic induction medium (Osteolife LM-0023, LOT NO 05007) supplemented with 1% penicillin/streptomycin solution (Capricorn cat# PS-B, Cat no CP13-1019). Osteogenic differentiation medium was replaced with fresh media after every 3-5 days and experiments were terminated after 21 days.

### 2.6 Assessment of Osteogenic DifferentiationVon Kossa staining

The assessment of DPSCs differentiation into osteoblasts was determined by “Von Kossa” staining [10]. Briefly, after 14 and 21 days of induction, the osteogenic differentiation medium was discarded, and cells were fixed with 10% formalin for 15 minutes. Cells were stained using a commercially available Von Kossa staining kit (MASTER TEC STAIN KITS lot No. AAEWD005) according to manufacturer instructions. Stained cultures were observed by bright field microscopy and images were captured.

### 2.7 Alizarin red staining

In addition to Von Kossa Staining, the osteogenic induced cultures were also stained with alizarin red S (Cat no 22889). Differentiated DPSCs were fixed with 10% formalin for 15 minutes and were stained with 2% Alizarin Red S for 15-30 minutes. Cultures were then thoroughly washed with deionized water and viewed under light microscope.

### 2.8 Polymerase chain reaction (PCR)

To evaluate the expression of lineage specific genes, polymerase chain reaction was performed. Briefly, RNA was isolated after day 14 and 21 of osteogenic induction by TRIZOL (Invitrogen) and cDNA was synthesized using reverse transcriptase (Wizscript cDNA synthesis kit). For PCR, WizPure™ PCR 2X Master Mix was used. Sequences for the primer pairs and their product lengths (bp) are given in Table 1 [10].

**Table 1:** List of used primers and their sequences

Sr #	Genes markers	PCR product (bp)	PCR primer set (5’-3’)
1	Beta actin	**137**	Forward primer: CGCATGGGTCAGAAGGATTC
			Reverse primers: TAGAAGGTGTGGTGCCAGATTT
2	OCN	**229**	Forward primer: CTCACACTCCTCGCCCTATT
			Reverse primer: CCTCCTGCTTGGACACAAA
3	RUNX	**250**	Forward primer: ACCTTGACCATAACCGTCTTCAC
			Reverse primer: TCCCGAGGTCCATCTACTGTAAC

The following PCR conditions were used; denaturation for 5 minutes at 95°C; followed by annealing for 30 seconds at 55°C and extension at 72°C. PCR products were visualized by agarose gel electrophoresis.

### 2.9 Data analysis

Statistical analysis was performed using GraphPad Prism 6 (Graphpad Software, Inc). Plating efficiency, population doublings and doubling time were presented as mean ± standard deviation.

## 3 Results

### 3.1 Isolation and Characterization of DPSCs

**Figure 1:**
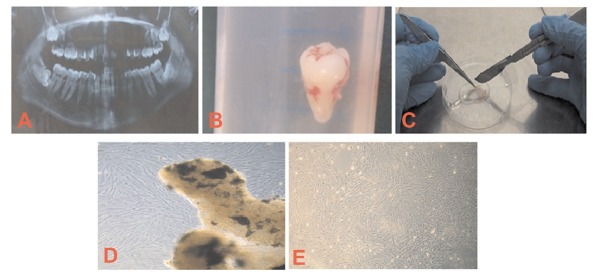
OPG, extract tooth and explant culture. A) An OPG showing impacted tooth. B) Extracted tooth in PBS. C) Pieces of pulp. D) MSCs started coming out of small DP pieces at 6-10 days. DP-MSCs at approximately 2 weeks after culture E) Cells formed monolayer after 14-21 days. OPG: orthopentomogram, DP: Dental pulp; MSCs: Mesenchymal stem cells.

MSCs from human dental pulp were successfully isolated from the pulp of third molars from all samples (n=6). After 4-6 days in culture, cells started to grow out of the small tissue segments and attached to the plastic surface. After approximately two weeks of culture, adherent cells became 70%-80% confluent and formed a monolayer within three weeks as shown in Figure 1. MSCs were characterized by their plastic adherent properties and fibroblastic morphology.

### 3.2 Growth Characteristics of Dental Pulp Stem Cells

**Figure 2:**
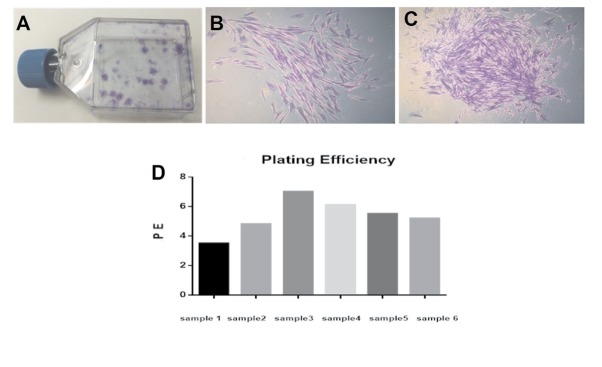
DPSC derived colonies and plating efficiency. A) showing colonies (low and high density) derived from DPSCs after 14 days. Colonies were stained with crystal violet dye. B) Low density colony of DPSCs and C) showing high density colony of DPSCs. D) Shows plating efficiency of all samples with different ages (n=6).

Growth characteristics of DPSCs were defined by parameters such as plating efficiency, number and time of population doublings. Plating efficiency was performed to evaluate the clonal expansion capacity of DPSCs. DPSCs grew into colonies with different densities within two weeks (Figure 2A). DPSCs from all donors produced colonies of different sizes (Figure 2B, C), and exhibited high plating efficiency (5.35% ± 1.18) as demonstrated in Figure 2D.

**Figure 3:**
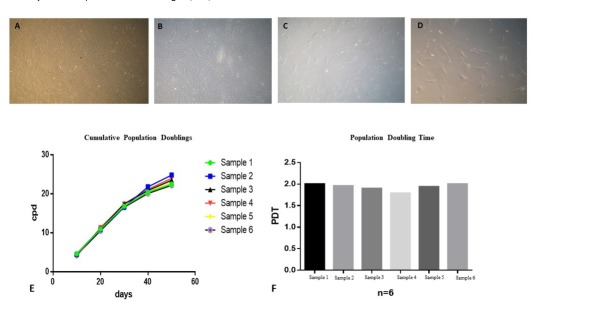
Proliferative potential of DPSCs. Passaging of DPSCs A) passage 1 B) passage 3 C) passage7 D) passage 10 E) cumulative population doublings of all six samples with different age groups from p0 to p 10 F) population doubling time of DPSCs was calculated for all six samples from P0- P10 , which was 1.95days ± 0.080, DP-MSCs shows an ideal PDT proving that these cells are easily expendable.

When cultured DPSCs reached 80-90% confluence, they were trypsinized and serially passaged at 1:10 dilution to determine cumulative population doublings up to passage ten. DPSCs showed excellent proliferative potential and expanded rapidly in culture. The number of cPDs of DPSCs culture for ten passages was 24.38 ± 1.11 (Figure 3E). Similarly, the population doubling time of DPSCs was 1.95 ± 0.080 days as shown in figure 3F.

### 3.3 Osteogenic Differentiation of DPSCs

**Figure 4:**
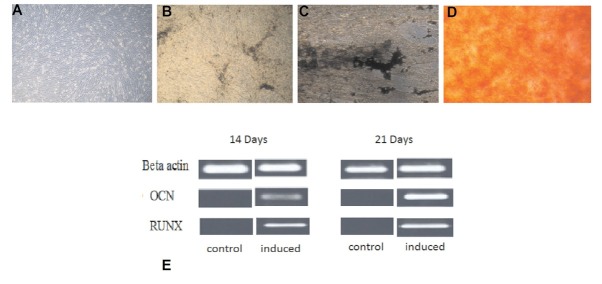
Differentiation potential of DPSCs. A) controlled, B, C) Von kossa staining at day 14 and 21 respectivly, confirming mineral deposition by newly formed osteoblasts D) alizarin red staining at day 21 E) showing positive results of RUNX2 and OCN at day 14 and 21 of osteogenic differentiation through polymerase chain reaction.

In osteogenic induction medium, the morphology of DPSC was changed from fibroblast to polygonal. After 21 days of induction, newly differentiated osteoblasts grew in multiple layers and stained positive for “Von Kossa” as well as “Alizarin red”, confirming the differentiation of DPSCs into osteoblasts. Positive RUNX2 expression was also observed at day 14 and 21, and significant up regulation of osteocalcin (OCN) was also observed after day 14 as shown in figure 4. RUNX2 transcription factor is known to appear at the initiation of osteogenic differentiation, and is considered the earliest and most precise marker for bone formation. The OCN protein marker is considered a late phase marker of osteogenic differentiation.

## 4 Discussion

The use of MSCs for regenerative medicine has garnered a significant consideration in recent years. Already, bone marrow stromal stem cells, adipose derived stem cells, cord blood and cord tissue have shown to be potent sources of MSCs [14, 15, 16, 17]. For the regeneration of bone, three components are required, namely; cells, scaffolds and growth factors [2]. Until now, BMSCs have been extensively exploited for osteogenic research and bone tissue restoration, but limited differentiation competence and proliferation potential makes it a less ideal source for MSCs [1, 2]. DPSCs possess a high proliferative potential and their source is readily available. Consequently, extracted wisdom teeth can serve as an ideal source of MSCs for tissue engineering. The development of the third molar teeth is a unique organogenesis event that occurs after the birth, being the last tooth to be developed at the age of six. Up until this point, prior to development initiation, embryonic tissue from the dental lamina remains dormant and undifferentiated with in the jawbones. Completion of crown formation occurs between the ages of 12-16 years and root development is completed at 18-25 years. These teeth can provide an optimal quality of dental pulp tissue with the presence of more cells and less fibers [4]. The main goal of this study was to isolate DPSCs from the dental pulp of healthy molars and differentiate these cells into osteoblasts. DPSCs can be isolated either by enzymatic digestion of pulp or by explant culture method [18, 20]. We selected explant culture method as it is inexpensive and gives a pure population of stem cells [21]. Within 3-4 days after primary culture, a certain number of cells (DPSCs) appeared in culture that resembled MSCs as defined by their spindle shape, fibroblastic morphology and plastic-adherent growth as documented previously [22, 23]. The International Society for Cellular Therapy (ISCT) defines the criteria for characterization of MSCs. According to this criteria, these cells exhibit positive expression of certain markers such as CD44, CD73, CD90, CD146 and CD166 while they are negative for hematopoietic lineage markers such as CD14, CD19, CD34 and CD45, as were exhibited by cells from other MSC sources [21, 22, 23]. Studies indicate that cells isolated by explant culture method are pure as compared to cells isolated by enzymatic digestion. DPSCs were isolated from the 3rd molars of donors of different ages. To evaluate growth characteristics, the cultures of DPSCs at 80%-90% were serially passaged up to passage 10. All resultant cultures of DPSCs showed high proliferative ability as indicated by their high number of population doublings (24.38 ±1.11) and low population doubling time (1.95 days ± 0.080). These results are in agreement with other similar studies by Huang et al [23], Suchánek J et al [24], Gronthos et al [22] and Kellner M et al [6]. DPSCs also exhibited high plating efficiency (5.35 ± 1.188), similar to the findings of Eslaminejad et al [25]. Stem cell numbers in dental pulp tissue when evaluated alongside other MSCs, i.e. bone marrow or adipose tissue, were shown to be less differentiated, bearing greater CFU-F as described by Gronthos et al [22] Huang et al [23] and El-Gendy et al [1]. The high proliferative potential of DPSCs indicate their potential use in applications such as tissue engineering and regenerative medicine.

Like MSCs from other sources, DPSC cells bear the capability to differentiate into multiple cell lineages under suitable in vitro conditions. In this *in vitro* experimental study, when DPSCs were cultured in media supplemented with dexamethasone, sodium β-glycerphosphate and L- ascorbic acid 2- phosphate for 21 days, they exhibited excellent differentiation into osteoblasts. We observed significant morphological changes from fibroblastic towards more plump and cuboidal shaped cells in induced cultures. In addition, newly differentiated osteoblasts formed organized ECM with calcium rich deposits in *in vitro* cultures that were detected by positive staining with “Von Kossa” and Alizarin Red. Results were similar to the findings documented by Graziano et al [26] and Kermani et al [13]. Expression of specific bone-forming gene markers was confirmed by PCR at day 14 and 21, to evaluate DPSC differentiation at the mRNA level. Over a period of 21 days, the transcription factor RUNX2 and osteocalcin (OCN) were up regulated in the monolayer of DPSCs under osteogenic conditions as normalized to control cultures of stem cell culture medium. The presence of RUNX2 was detected early during osteoblast differentiation, as an early osteogenesis marker at day 14, and was considered an important signal during osteoblast differentiation from DPSCs. RUNX2 also activates the expression of OCN which is a specific indicator of osteogenic differentiation. Previous, data indicated that OCN is produced only at the termination of osteogenic matrix maturation by matured osteoblasts, however, its presence can be detected as early as day 14 of osteogenic differentiation. These results were in line with other studies [27, 28, 29, 30].

Stem cell based bone tissue engineering holds a promising prospect in the treatment of medical and dental conditions [28, 29, 30]. Currently, in Pakistan, there is an increase in reported cases of trauma due to high-speed vehicle, bomb blast, violence, and cancers of the maxillofacial region. This study will help to lessen the resultant disabilities and promote the normalization of patients with better acceptance and functionality in the society. Excellent growth characteristics and osteogenic differentiation potential of human dental pulp stem cells *in vitro* suggests that DPSCs could be used for therapeutic purposes where restoration of bone is a challenge. Cryopreservation of autologous DPSCs would allow the banking of stem cells in anticipation with future therapies. In conclusion, the impacted 3^rd^ molar is a rich and easily accessible source of DPSCs. Isolated DPSCs possess a high proliferative potential and ability to differentiate into osteoblasts and therefore could be used for regenerative medicine applications in oral and maxillofacial surgery.

## References

[1] El-Gendy R, Yang XB, Newby PJ, Boccaccini AR, Kirkham J (2013). Osteogenic Differentiation of Human Dental Pulp Stromal cells on 45S5 BioglassR Based Scaffolds in Vitro and In Vivo. Tissue Eng.

[2] Ito K, Yamada Y, Nakamura S, Ueda M (2011). Osteogenic Potential of Effective Bone Engineering Using Dental Pulp Stem Cells, Bone Marrow Stem Cells and Periosteal Cells for Osseo integration of Dental Implants. Int J Oral Maxillofac Implants.

[3] Lizier NF, Kerkis A, Gomes CM, Hebling J, Oliveira CF, Caplan AI (2012). Scaling-up of dental pulp stem cells isolated from multiple niches. PLOS One.

[4] Vishwanath VR, Nadig RR, Nadig R, Prasanna JS, Karthik J, Pai VS (2013). Differentiation of isolated and characterized human dental pulp stem cells and stem cells from human exfoliated deciduous teeth: An in vitro study. J Conserv Dent.

[5] Didilescu AC, Rusu MC, Nini G (2013). Dental pulp as a stem cell reservoir. Rom J Morphol Embryol.

[6] Kellner M, Steindorff MM, Strempel JF, Winkel A, Kuhnel MP, Stiesch M (2014). Differences of isolated stem cells depend on donor age and the consequences for autologous tooth replacement. Arch Oral Biol.

[7] Ma L, Makino Y, Yamaza H, Akiyama K, Hoshino Y, Song G (2012). Cryopreserved dental pulp tissues of exfoliated deciduous teeth is a feasible stem cell resource for regenerative medicine. PLoS One.

[8] Tatullo M, Marrelli M, Shakesheff KM, White LJ (2015). Dental pulp stem cells: function, isolation and application in regenerative medicine. J Tissue Eng Regen Med.

[9] Shaikh RA (2013). Therapeutic Potential of Stem Cells in Regenerative Dentistry; a Review of Literature. IDJSR.

[10] Li JH, Liu DY, Zhang FM, Wang F, Zhang WK, Zhang ZT (2011). Human dental pulp stem cell is a promising autologous seed cell for bone tissue engineering. Chin Med J.

[11] d’Aquino R, De Rosa A, Lanza V, Tirino V, Laino L, Graziano A (2009). Human mendible bone defect repair by the grafting of dental pulp stem/progenitor cells and collagen sponge biocomplexes. Eur Cell Mater.

[12] Huang CE, Hu FW, Yu CH, Tsai LL, Lee TH, Chou MY (2014). Concurrent expression of oct4 and nanog maintains mesenchymal stem-like property of human dental pulp cell. Int J MolSci.

[13] Kermani S, Megat Abdul Wahab R, Zarina Zainol Abidin I, Zainal Ariffin Z, Senafi S, Hisham Zainal Ariffin S (2014). Differentiation Capacity of Mouse Dental Pulp Stem Cell into Osteoblasts and Osteoclasts. Cell J.

[14] Choudhery MS, Badowski M, Muise A, Harris DT (2013). Utility of cryopreserved umbilical cord tissue for regenerative medicine. Current Stem Cell Research & Therapy.

[15] Choudhery MS, Badowski M, Muise A, Harris DT (2014). Cryopreservation of whole adipose tissue for future use in regenerative medicine. Journal of Surgical Research.

[16] Mahmood R, Choudhery MS, Mehmood A, Khan SN, Riazuddin S (2015). In vitro differentiation potential of human placenta derived cells into skin cells. Stem Cells International.

[17] Fatima Q, Chaudhry N, Choudhery MS (2018). Umbilical cord tissue derived mesenchymal stem cells can differentiate into skin cells. Open Life Sciences.

[18] Kim BC, Bae H, Kwon IK, Lee EJ, Park JH, Khademhosseini A (2012). Osteoblastic/Cementoblastic and Neural Differentiation of Dental Stem Cells and Their Applications to Tissue Engineering and Regenerative Medicine. Tissue Engineering Part B: Reviews.

[19] Gronthos S, Mankani M, Brahim J, Robey PG, Shi S (2000). Postnatal human dental pulp stem cells (DPSCs) in vitro and in vivo. Proc Natl Acad Sci USA.

[20] Ricordi C, Tzakis AG, Carroll PB, Zeng YJ, Rilo HL, Alejandro R (1992). Human islet isolation and allotransplantation in 22 consecutive cases. Transplantation.

[21] Choudhery MS, Badowski M, Muise A, Harris DT (2013). Comparison of human mesenchymal stem cells derived from adipose and cord tissue. Cytotherapy.

[22] Farahzadi R, Fathi E, Mesbah-Namin SA, Zarghami N (2018). Anti-aging protective effect of L-carnitine as clinical agent in regenerative medicine through increasing telomerase activity and change in the hTERT promoter CpG island methylation status of adipose tissue-derived mesenchymal stem cells. Tissue Cell.

[23] Fathi E, Farahzadi R, Valipour B, Sanaat D (2019). Cytokines secreted from bone marrow derived mesenchymal stem cells promote apoptosis and change cell cycle distribution of K562 cell line as clinical agent in cell transplantation. PLoS One.

[24] Suchánek J, Soukup T, Ivancaková R, Karbanová J, Hubková V, Pytlík R (2007). Human dental pulp stem cells--isolation and long term cultivation. Acta Medica (Hradec Kralove).

[25] Eslaminejad MB, Vahabi S, Shariati M, Nazarian H (2010). In vitro Growth and Characterization of Stem Cells from Human Dental Pulp of Deciduous Versus Permanent Teeth. J Dent (Tehran).

[26] Graziano A, d’Aquino R, Laino G, Papaccio G (2008). Dental pulp stem cells: a promising tool for bone regeneration. Stem Cell Rev.

[27] Hilkens P, Gervois P, Fanton Y, Vanormelingen J, Martens W, Struys T (2013). Effect of isolation methodology on stem cell properties and multilineage differentiation potential of human dental pulp stem cells. Cell Tissue Res.

[28] d’Aquino R, Graziano A, Sampaolesi M, Laino G, Pirozzi G, De Rosa A (2007). Human postnatal dental pulp cells co-differentiate into osteoblasts and endotheliocytes: a pivotal synergy leading to adult bone tissue formation. Cell Death Differ.

[29] Wang Y, Yan M, Wang Z, Wu J, Wang Z, Zheng Y (2013). Dental pulp stem cells from traumatically exposed pulps exhibited an enhanced osteogenic potential and weakened odontogenic capacity. Arch Oral Biol.

[30] Bressan E, Ferroni L, Gardin C, Pinton P, Stellini E, Botticelli D (2012). Donor age-related biological properties of human dental pulp stem cells change in nanostructured scaffolds. PLoS One.

